# AD Workbench: Transforming Alzheimer's research with secure, global, and collaborative data sharing and analysis

**DOI:** 10.1002/alz.70278

**Published:** 2025-05-19

**Authors:** Caitlin P. McHugh, Matthew H. S. Clement, Mukta Phatak

**Affiliations:** ^1^ Alzheimer's Disease Data Initiative Kirkland Washington USA

**Keywords:** Alzheimer's disease, cloud‐computing analysis, data sharing

## Abstract

**INTRODUCTION:**

The Alzheimer's Disease Data Initiative (AD Data Initiative) is a global coalition of partners accelerating scientific discoveries in Alzheimer's disease (AD) and related dementias (ADRD) by breaking down data silos, eliminating barriers to research, and fostering collaboration among scientists studying these issues.

**METHODS:**

The flagship product of the AD Data Initiative technical suite is AD Workbench, a secure, cloud‐based environment that enables global access, analysis, and sharing of datasets, as well as interoperability with other key data platforms.

**RESULTS:**

As of April 7, 2025, AD Workbench has 6178 registered users from 115 countries, including 886 users from 60 low‐ and middle‐income countries. On average, more than 500 users, including over 100 new users, log in each month to discover data and conduct integrative analyses.

**DISCUSSION:**

By prioritizing interoperability and robust security within a collaborative framework, AD Workbench is well positioned to drive advancements in AD treatments and diagnostic tools.

**Highlights:**

- Data sharing
- Interoperability
- Cloud‐based analytics
- Collaborative workspace

## 1 INTRODUCTION

The Alzheimer's Disease Data Initiative (AD Data Initiative)^1^ is a global nonprofit coalition of partners aimed at accelerating scientific discoveries in Alzheimer's disease (AD) and related dementias (ADRD). Founded in 2020 by a group of leading research, health, and advocacy organizations involved in combatting AD, the coalition recognized the value in existing data but acknowledged the limited statistical power of study data shared at the time, often residing in disparate repositories. They committed to creating a solution to galvanize researchers with as few barriers as possible, which resulted in developing a free resource linking data discovery, access, permissions, and analysis. The AD Data Initiative's mission is to accelerate scientific breakthroughs in AD research by expanding access to relevant data.

**FIGURE 1 alz70278-fig-0001:**
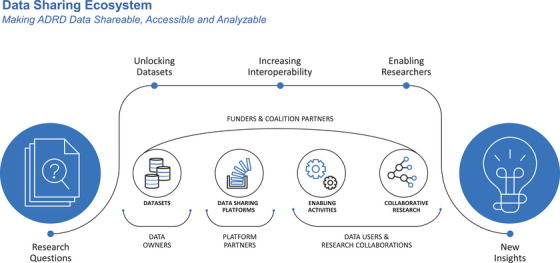
AD Data Initiative data sharing ecosystem.

To achieve this, the AD Data Initiative breaks down data silos and eliminates barriers inhibiting fruitful research partnerships by facilitating secure data sharing, fostering collaboration, and integrating advanced analytical tools (Figure 1). The flagship offering is AD Workbench, a secure, cloud‐based data sharing and analytics environment that empowers researchers with access to diverse datasets and powerful analytic capabilities in a collaborative, interoperable environment. Today, the AD Data Initiative coalition members help guide, advise, and advance scientific research through participation on the organization's advisory board.^2^ In addition, the coalition members help unlock data not currently shared and support the generation of data needed to address key unanswered questions.

The AD Workbench functions as an interoperable layer connecting data platforms and repositories around the world to support researchers with free compute, the opportunity to combine data, and to collaborate. It simplifies data gathering while allowing for a wider net to be cast for useful data. It helps reduce the transfer and storage of redundant dataset copies, which generate significant unnecessary expenses. The AD Workbench makes it easier for geographically dispersed researchers to work together and generate analytic insights from wide‐ranging scientific sources, thus strengthening efforts to develop effective treatments, diagnostics, and ultimately a cure for AD.

## 2 METHODS

The AD Workbench comprises two core components: (1) a findable, accessible, interoperable, and reusable (FAIR)–compliant^3^ data catalog for dataset discovery with a streamlined, customizable data access request system; and (2) secure, collaborative online workspaces for conducting analyses. This integrated design enables users to search and request datasets from diverse global sources and conduct integrative analyses in a secure environment.

**RESEARCH IN CONTEXT**

1. **Systematic review**: The authors are part of the Alzheimer's Disease Data Initiative (AD Data Initiative), which developed AD Workbench based on collaborative discussions with coalition members and key stakeholders, including scientists, on their primary research needs. They also reviewed the workings of other data repositories in Alzheimer's and related spaces, such as the Global Alzheimer's Association Interactive Network (GAAIN) and the Dementias Platform UK (DPUK) Data Portal.
2. **Interpretation**: At more than 6100 users, AD Workbench has grown into a key collaborative platform for Alzheimer's research and diagnostics development that enables geographically disparate teams to share data and perform analyses in a secure cloud‐based environment.
3. **Future directions**: The AD Data Initiative team is actively expanding AD Workbench with interoperable data offerings and advanced functionalities, aiming to foster collaboration. Planned features include the introduction of secure enclaves to protect shared data and cutting‐edge generative artificial intelligence (AI) tools to revolutionize data analysis.

*AD Discovery Portal*: AD Workbench's publicly accessible catalog of datasets is designed in accordance with the National Institutes[Fig alz70278-fig-0001] of Health (NIH) Data Management and Sharing policies and the FAIR Guiding Principles for scientific data management, which maximize the value of research data, make it easier to find, and encourage its reuse.^4^ The AD Discovery Portal includes the following features:

1. Data type‐agnostic: AD Workbench's robust infrastructure supports various data types, allowing researchers to request access to multimodal data, such as clinical, genomic, and imaging, that is essential for understanding complex diseases like AD. Although agnostic to data type, anonymizing data at its source is a prerequisite to sharing data on the platform.
2. Customizable data access requests: Data access requests can be customized to align with specific governance requirements. Data providers retain full control over the process, deciding whether to approve or reject access requests.
3. Reporting capabilities: Data usage visibility provides insights into who requested data and their intended research purpose.
4. Extensive metadata for discovery: The catalog includes comprehensive metadata and a data dictionary to help researchers find relevant datasets based on facets and/or fuzzy search and assess dataset suitability.
5. Flexible data sharing models: AD Workbench offers several data‐sharing models to accommodate diverse governance needs. The centralized model stores data within the AD Data Initiative repository for direct data sharing, whereas the distributed model keeps data at its source, transferring only select record‐level data to AD Workbench workspaces; in the federated model, data remain entirely at the source, with remote querying and results released post‐analysis.
6. Version control and flexible data ingestion: Version control options and flexible data transfer methods are provided for seamless data integration.

*Workspaces*: Once researchers have discovered and been granted access to relevant data, analyses proceed via workspaces, which are secure, standards‐compliant environments for collaborative research. AD Workbench's governance model ensures strict data protection by limiting download permissions, maintaining comprehensive audit trails, and ensuring privacy and control over sensitive datasets, optionally with an administrator overseeing file exports to further enforce security. In addition, AD Workbench is designed to meet high data security standards, including the General Data Protection Regulation (GDPR)^5^ and the Health Insurance Portability and Accountability Act (HIPAA).^6^ AD Workbench offers the AD Curation Studio, an environment for anyone who needs to remove sensitive information from a dataset before sharing.

### 2.1 Significant features of AD Workbench's workspaces

*Global hubs*: Workspaces can be deployed across geographic hubs to meet varied governance and compliance requirements, including GDPR and HIPAA standards.

*Pre‐built analytical tools*: Workspaces include tools for common bioinformatics analyses such as R,^7^ Python, and Jupyter Notebooks, reducing configuration time and allowing researchers to focus on analysis.

*Self‐service customization*: Researchers can upload their own data and tools, tailor the platform to fit their specific needs, and adjust workspace configurations, as necessary.

*Scalability and virtual infrastructure*: AD Workbench provides scalable virtual machines (VMs) for high‐capacity computing, deployed with essential tools to accelerate research and enhance productivity. Storage and computing resources are available in‐kind with a pre‐built configuration, including 5TB of storage and four virtual central processing units (CPUs) with 14GB of random access memory (RAM). Researchers can request additional resources based on project needs.

*Airlock and audit trail*: Datasets in workspaces are kept secure with an airlock feature that ensures nothing is shared outside the project until the team administrator decides that the analysis is ready. An audit trail keeps tabs on actions taken by each user.

### 2.2 Unique value propositions of AD Workbench

AD Workbench is designed with several unique value propositions that amplify its utility for both users and data‐sharing partners, including:

*A diverse, global user base*: AD Workbench addresses traditional data‐sharing barriers by providing an inclusive platform for those sharing datasets and those analyzing data, cultivating a cohesive research community under one ecosystem. By broadening access to data, AD Workbench supports a wide array of researchers globally.

*Global collaboration and community support*: Beyond data access, AD Workbench enables researchers to share code, methodologies, and insights, thereby enhancing collaborative research efforts. The AD Data Initiative coalition also fosters an active community with events like data challenges and the flourishing William H. Gates Sr. Fellowship program, fueling innovation and engagement in Alzheimer's research.

*Collaborative workspaces*: AD Workbench offers role‐based workspace access, allowing administrators to manage memberships, which is ideal for consortiums. Workspaces also support use cases like anonymous manuscript review, providing dataset access while preserving anonymity.

*Flexible data sharing options*: AD Workbench allows data providers to customize data sharing based on governance requirements, with options for data access, download permissions, and embargo periods. This adaptability ensures compliance with various sharing policies.

*Brand‐preserving model*: Data providers maintain ownership, visibility, and control over their datasets. With customizable data access forms and approval workflows, providers maintain autonomy over how their data are accessed and used.

*Secure data access with federated data sharing*: The AD Data Initiative has developed and integrated the Federated Data Sharing Appliance (FDSA)^8^ into AD Workbench, which enables researchers to conduct analyses without direct data access, with data providers approving results release. This model ensures data privacy and governance while supporting collaborative research.

*Regulatory compliance via Curation Studio*: Although AD Workbench adheres to HIPAA and GDPR standards, the Data Contributor's Agreement requires data to be anonymized at its source before sharing. However, data providers sometimes need infrastructure support for curation and preprocessing tasks. The “Curation Studio” lets providers securely upload sensitive data, including protected health information (PHI) and personally identifiable information (PII), for these processes. After preparation, data can then be shared publicly via AD Workbench.

*DOI minting service*: AD Workbench offers a free digital object identifier (DOI) minting service, providing unique identifiers for datasets to support research reproducibility and citation.

*Ecosystem of platforms*: AD Workbench, acting as the interoperability layer, enables researchers to integrate datasets from different sources, which is crucial for large‐scale, cross‐disciplinary ADRD research. AD Workbench offers interoperability and/or Single Sign‐On (SSO) with other platforms focused on ADRD, including:

*American Heart Association's Precision Medicine Platform (PMP)*: A platform that houses more than 13,000,000 national patient records.^9^
*Answer ALS Neuromine*: A portal with multimodal data on over 1250 participants.^10^
*Critical Path for Alzheimer's Disease (CPAD) database*: A subset of the 36 AD/MCI clinical trial placebo arm data is available via a distributed connection.^11^
*Dementias Platform UK (DPUK) Data Portal*: A collaborative environment for dementia research, providing access to diverse data types and analytical tools.^12^
*European Platform for Neurodegenerative Diseases (EPND) Technical Hub*: A centralized catalog offering biosample and dataset discovery and access to over 250,000 participants from 95 studies.^13^
*Global Alzheimer's Association Interactive Network (GAAIN)*: A network offering access to a vast array of Alzheimer's data, integrating datasets from international sources and facilitating analysis and visualization for global collaboration.^14^
*Health Data Research (HDR) UK Gateway*: A search and request portal for UK health data. AD Data Initiative users have federated access for metadata discovery.^15^
*Vivli*: A global data‐sharing platform that provides researchers with access to anonymized clinical trial data.^16^ ADRD‐specific datasets on this platform are interoperable with AD Workbench.

The AD Data Initiative continues to expand its network of interoperable platforms, for example, we are partnering with Sage Bionetworks to link AD Workbench and the NIH‐supported AD Knowledge Portal,^17^ which will introduce over 140 additional datasets crucial for advancing research.

## 3 RESULTS

As of April 7, 2025, the AD Workbench has 6178 registered users from 115 countries, including 886 users from 60 low‐ and middle‐income countries. On average, more than 500 users, including over 100 new users, log in each month.

**FIGURE 2:**
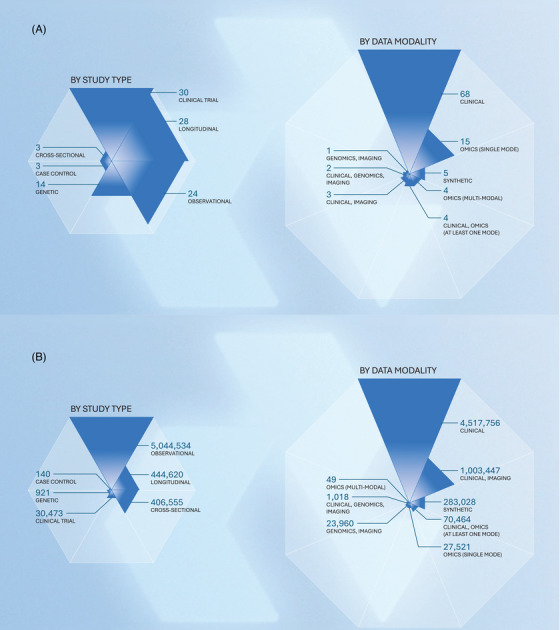
Data currently available on the platform. (A) 102 datasets, by study type and data modality. (B) 5,927,243 participant records, by study type and data modality.

In addition, AD Workbench currently enables access (with permission) to over 5,800,000 participant records from 105 studies (Table S1). Datasets accessible via AD Workbench include interventional trials, longitudinal studies, and encompass numerous data modalities (Figure 2). The studies come from 69 data contributors, and users have submitted 842 data access requests, of which 623 have been approved. This 74% approval rate indicates that the platform is cultivating a culture of partners committed to sharing their data.

The unique infrastructure design of AD Workbench has enabled researchers to interrogate key questions in ADRD. For example, workspaces have been leveraged to pursue the following studies:

*Exploring AD heterogeneity*: Researchers are investigating AD heterogeneity, particularly through factors like amyloid composition and differential protein expression across brain regions.

*Testing diagnostic and predictive models*: Workspaces have been created to develop models that may improve AD diagnostics, assess model performance, and create explainable AI models.

*Studying environmental and demographic factors*: Projects focus on understanding AD prevalence in underrepresented populations and the influence of environmental conditions.

*Omics data analysis*: Several groups of researchers are analyzing omics data to understand disease mechanisms, identify biomarkers, and assess genetic contributions to AD progression.

*Pilot testing and validation*: Workspaces are being used to test and validate newly generated datasets and tools in preparation for larger‐scale studies.

Several data hackathons have been led by AD Data Initiative. One such event was a virtual NeuroToolKit (NTK) hackathon held from July 1 to 17, 2022.^18^ This multi‐institution research effort brought together 45 multi‐person teams from 11 countries. The event leveraged a dataset accessible via AD Workbench, the collaborative workspaces, and pre‐configured analysis tools to interrogate how the amyloid, tau, and neurodegeneration (ATN) criteria are associated with various profiles in high‐risk individuals. At least one team has published their work from that hackathon.^19^

Another large‐scale project taking advantage of AD Workbench is the EPND, which aims to address challenges in biomarker research via a centralized discovery and access platform.^13^ EPND is leveraging AD Workbench as the key infrastructure to facilitate its mission, help researchers navigate legal and ethical requirements, and create a faster, more collaborative, and more disruptive research environment for ADRD diagnostics and therapies via SSO with the EPND Hub. Such consortium projects exemplify how AD Workbench can provide critical support and significant value to funding bodies that are interested in sponsoring collaborative research.

As a coalition of partners offering a data sharing platform, the AD Data Initiative is also in an exceptional position to share and map commonly used ontologies. Rather than require data sharing groups to format their data in a prescribed manner, AD Workbench makes available a mapping of ADRD research variables across three commonly used ontologies, including the C‐SURV ontology.^20^ With community involvement, this mapping can expand to any number of standards and help researchers quickly map variables to their preferred ontology. Datasets on AD Workbench not currently mapped are soon to be harmonized by the AD Data Initiative to accelerate analysis, reduce opportunities for error, and diminish redundant data.

## 4 DISCUSSION

By offering a secure, scalable, and collaborative environment for global data sharing and analysis, AD Workbench is poised to become a transformative platform in Alzheimer's research. AD Workbench enables researchers worldwide to securely access, analyze, and share datasets, thus breaking down traditional data silos and accelerating breakthroughs. By prioritizing interoperability and robust security within a collaborative framework, AD Workbench provides an invaluable resource for the research community and is positioned to drive advances in AD treatments and diagnostic tools. The emphasis on interoperability while providing an integrated infrastructure and in‐kind compute resources is the primary, unique differentiator of AD Workbench.

The members of the AD Data Initiative coalition are now working to further improve AD Workbench's offerings and help platform's users overcome still‐extant data challenges. For example, although AD Workbench's federated sharing model enables access to previously unreachable datasets, limited direct data access can still inhibit collaboration. To address this, the AD Data Initiative is pursuing a Secure Enclave Solution: a secure, cloud‐based environment that offers direct access to approved datasets while allowing data providers to retain full control. Key features of this enclave may include:

*Trusted Execution Environments (TEE)*: Ensuring secure execution of research tasks.

*Data protection without downloads*: Allowing research results to be exported while keeping record‐level data protected.

*Federated data governance*: Data providers maintain control as gatekeepers over their data.

*Time‐bound environments*: Configurable for specific projects or durations, as necessitated by collaborators.

The secure enclave model will enhance AD Workbench's utility by unlocking restricted datasets for integrated research and enabling secure, direct access. By facilitating integrative studies and empowering researchers to leverage a more connected dataset landscape, this approach could further accelerate the journey toward new discoveries and advancements.

Looking forward, AD Workbench also aims to expand its impact by integrating more AI and generative AI tools into the platform. Key potential enhancements of an AI‐powered research workbench include:

- A generative AI chatbot for data discovery, cataloging, and tailored dataset recommendations.
- On‐demand cross‐cohort variable searches leveraging harmonized metadata schemas.
- AI copilot integration for workspace code support.
- Synthetic data generation for restricted datasets.
- GPU infrastructure for complex AI and Gen AI workloads.
- Improved efficiency and quality of service desk operations.

Through these and further enhancements, AD Workbench will continue to push Alzheimer's research forward and expand the boundaries of what is possible.

## CONFLICT OF INTEREST STATEMENT

The authors declare no conflict of interest. Author disclosures are available in the Supporting Information.

## Supporting information



Supporting Information

Supporting Information
