# Supplementary material for: AD Workbench: Transforming Alzheimer's research with secure, global, and collaborative data sharing and analysis
Source: Alzheimers Dement. 2025 May 19;21(5):e70278. doi: 10.1002/alz.70278 (PMC12086970; doi:10.1002/alz.70278)
Supplement: Supplementary file 2 — Supporting Information [file ALZ-21-e70278-s001.docx]

Supplementary Table 1. Datasets accessible through AD Discovery Portal, alphabetical by Title.

| **Title** | **Count of Participants** | **Study Types** | **Data Types** |
| --- | --- | --- | --- |
| A blood based 12-miRNA signature of Alzheimer patients - GSE46579 | 70 | Genetic | Omics (single mode) |
| A dataset of EEG recordings from: Alzheimer's disease, Frontotemporal dementia and Healthy subjects | 88 | Observational | Clinical |
| A double-blind, randomised, placebo-controlled, parallel-group dose-ranging study to investigate the effects of rosiglitazone on cognition in subjects with mild to moderate Alzheimer's disease. | 500 | Clinical Trial | Clinical |
| A Phase 2a Study to Evaluate the Effect of Rilapladib (SB-659032) on Biomarkers Related to the Pathogenesis and Progression of Alzheimer's Disease | 124 | Clinical Trial | Clinical |
| A Randomised, Double-blind, Placebo-controlled Study to Evaluate the Efficacy and Safety of the H3 Receptor Antagonist, GSK239512 in Subjects With Mild to Moderate Alzheimer's Disease. | 196 | Clinical Trial | Clinical |
| A Randomised, Single-Blind, Placebo-Controlled Study to Investigate the Safety, Tolerability, Immunogenicity, Pharmacokinetics and Pharmacodynamics of Intravenous Infusion of GSK933776 in Patients With Alzheimer's Disease. | 50 | Clinical Trial | Clinical |
| A reference induced pluripotent stem cell line for large-scale collaborative studies | 8 | Genetic | Omics (multi-modal) |
| A Single Blind, Placebo-controlled, Randomised Study in Mild to Moderate Alzheimer's Disease Patients to Assess the Safety, Tolerability, Pharmacokinetics and Pharmacodynamics of GSK239512, a Selective Histamine H3 Receptor Antagonist | 28 | Clinical Trial | Clinical |
| A Study of Semagacestat for Alzheimer's Patients | 180 | Clinical Trial | Clinical |
| Aberdeen Children of the 1950s ACONF | 12150 | Longitudinal | Clinical |
| AD Synthetic Patient Data | 5000 | Cross-sectional | Synthetic |
| ADDIA Alzheimer’s Disease Diagnostics Clinical Study | 800 | Clinical Trial | Clinical |
| Alzheimer's Disease and Healthy Aging Data | 250937 | Cross-sectional | Clinical |
| Alzheimer's prevention virtual cohort | 125000 | Observational | Synthetic |
| Amyloid imaging for Phenotyping Lewy body dementia (AMPLE) | 80 | Longitudinal | Clinical, Imaging |
| AMYPAD PNHS (Harmonised and Derived) v202306 | 3368 | Observational | Clinical, Imaging |
| An Open Label Single Oral Dose Study in Patients With Mild Alzheimer's Disease to Assess the Pharmacokinetics of Extended Release Formulation of Rosiglitazone (RSG XR) in This Population | 14 | Clinical Trial | Clinical |
| An open-label extension to study AVA100193, to assess the long-term safety and efficacy of rosiglitazone (extended release tablets) in subjects with mild to moderate Alzheimer's disease | 200 | Clinical Trial | Clinical |
| An Open-label Extension to Study AVA102670 and AVA102672, to Assess the Long-term Safety and Efficacy of Rosiglitazone (Extended Release Tablets) as Adjunctive Therapy on Cognition in Subjects With Mild to Moderate Alzheimer's Disease. | 1461 | Clinical Trial | Clinical |
| An Open-label Extension to Study AVA105640, to Assess the Long-term Safety and Efficacy of Rosiglitazone (Extended Release Tablets) on Cognition in Subjects With Mild to Moderate Alzheimer's Disease. | 331 | Clinical Trial | Clinical |
| Answer ALS | 1269 | Longitudinal | Clinical, Omics (at least one mode) |
| Banner Alzheimer's Prevention Initiative - Gen1(CAPI015) | 480 | Clinical Trial | Clinical |
| Banner Alzheimer's Prevention Initiative - Gen2(CNP520) | 1145 | Clinical Trial | Clinical |
| BCG-PANDA | 49 | Longitudinal | Clinical |
| BHDP - General Acute Inpatient and Day Case - Scottish Morbidity Record (SMR01) | 784200 | Observational | Clinical |
| BHDP - Mental Health Inpatient and Day Case - Scottish Morbidity Record (SMR04) | 62080 | Observational | Clinical |
| BHDP - National Records of Scotland (NRS) - Deaths Data | 350505 | Observational | Clinical |
| BHDP - Outpatient Appointments and Attendances - SMR00 | 820959 | Observational | Clinical |
| BHDP - Prescribing Information System (PIS) | 818360 | Observational | Clinical |
| BHDP - Scotland Accident and Emergency | 784200 | Observational | Clinical |
| BHDP - Scottish Cancer Registry (SMR06) | 219396 | Observational | Clinical |
| BLSA Open Data | 502 | Longitudinal | Clinical |
| BLSA Permissioned Data | 3453 | Longitudinal | Clinical |
| BLSA Synthetic Data | 2210 | Longitudinal | Synthetic |
| Boston University – Au Lab (Precision Brain Health Initiative) | 212 | Observational | Clinical |
| Brain Imaging Study Of Rosiglitazone Efficacy And Safety In Alzheimer's Disease | 80 | Clinical Trial | Clinical |
| Brain long read sequencing | 17 | Genetic | Omics (single mode) |
| Brains for Dementia Research | 3200 | Observational | Clinical |
| Bulk whole genome sequencing of cells derived from dermis and postmortem dura mater from the same subject | 1 | Genetic | Omics (single mode) |
| CamPaIGN Cohort Data | 142 | Clinical Trial | Clinical |
| Cognitive Function and Ageing Study | 18005 | Longitudinal | Omics (single mode) |
| Cognitive Function in Ageing Study II (CFAS II) | 7524 | Longitudinal | Clinical |
| Continued Efficacy and Safety Monitoring of Solanezumab, an Anti-Amyloid β Antibody in Patients With Alzheimer's Disease (EXPEDITION EXT) | 1457 | Clinical Trial | Clinical |
| CRISPRi Screens in iPSC derived neurons | 0 | Genetic | Omics (single mode) |
| Critical Path For Alzheimer's Disease (CPAD) Database | 12811 | Observational | Clinical |
| Dementia Research Group - Incidence Database | 14896 | Longitudinal | Clinical |
| Dementia Research Group 10/66 - Life2Years database | 6917 | Longitudinal | Clinical |
| Dementia Research Group 10/66 - Prevalence Database | 14896 | Longitudinal | Clinical |
| Detection of Alzheimer’s Disease at Mild Cognitive Impairment and Disease Progression Using Autoantibodies as Blood-based Biomarkers - GSE74763 | 100 | Genetic | Omics (single mode) |
| Digital Medicine Society Mixed Method Survey of Meaningful Aspects of Health in Alzheimer’s Disease and Related Dementias (ADRD). | 1007 | Observational | Clinical |
| DPUK Synthetic Dataset | 150618 | Cross-sectional | Synthetic |
| Effect of LY2062430, an Anti-Amyloid Beta Monoclonal Antibody, on the Progression of Alzheimer's Disease as Compared With Placebo (EXPEDITION) | 1000 | Clinical Trial | Clinical |
| Effect of LY450139 on the Long Term Progression of Alzheimer's Disease | 1537 | Clinical Trial | Clinical |
| Effect of Passive Immunization on the Progression of Alzheimer's Disease: LY2062430 Versus Placebo (EXPEDITION 2) | 1040 | Clinical Trial | Clinical |
| Effect of Passive Immunization on the Progression of Mild Alzheimer's Disease: Solanezumab (LY2062430) Versus Placebo (EXPEDITION 3) | 2129 | Clinical Trial | Clinical |
| Effects of LY450139 Dihydrate on Subjects With Mild to Moderate Alzheimer's Disease | 45 | Clinical Trial | Clinical |
| Effects of LY450139, on the Progression of Alzheimer's Disease as Compared With Placebo | 1111 | Clinical Trial | Clinical |
| EPAD LCS v.IMI | 2096 | Longitudinal | Clinical, Omics (at least one mode) |
| EPAD LCS v1500.0 | 1500 | Longitudinal | Clinical |
| EPAD LCS v500.0 | 500 | Longitudinal | Clinical |
| EPAD LCS v500.1 | 500 | Longitudinal | Clinical |
| EPND ATN study subset | 353 | Clinical Trial | Clinical |
| Five CSF proteomic subtypes in AD | 606 | Genetic | Omics (single mode) |
| Fox Insight | 54614 | Longitudinal | Clinical |
| Generation Scotland: Scottish Family Health Study | 23960 | Longitudinal | Genomics, Imaging |
| Genome-wide DNA methylation profiling in the superior temporal gyrus reveals epigenetic signatures associated with Alzheimer's disease - GSE76105 | 68 | Genetic | Omics (single mode) |
| GERAS Cohorts: Harmonized | 4328 | Longitudinal | Clinical |
| Harmonized 1066 MexCog Dataset | 16115 | Longitudinal | Clinical |
| Honolulu-Asia Aging Study | 3734 | Longitudinal | Clinical |
| Human iPSC Astrocytes and iMGL Transcripts | 1 | Genetic | Omics (single mode) |
| Mayo Clinic Study of Aging Clinical + PET Data | 533 | Longitudinal | Clinical |
| MindADmini | 93 | Clinical Trial | Clinical |
| Mis-spliced transcripts generate de novo proteins in TDP-43-related ALS/FTD | 15 | Genetic | Omics (multi-modal) |
| Modulation of Beta-amyloid Levels in CSF and Plasma by GSK933776 in Patients With Mild Alzheimer's Disease or Mild Cognitive Impairment | 19 | Clinical Trial | Clinical |
| Moorfields Eye Dementia Dataset | 232000 | Longitudinal | Clinical |
| Neurite and whole cell transcriptomics on iNeurons | 1 | Genetic | Omics (single mode) |
| Neuropathological quantifications, transcriptome, and in situ proteomics profile datasets for neuronal basis of circadian rhythm dysfunction in tauopathies | 10 | Case-control | Omics (multi-modal) |
| Northern Ireland Cohort for the Longitudinal study of Ageing (NICOLA) | 8504 | Longitudinal | Omics (single mode) |
| Open-Label Extension Assessing Long-Term Safety Of Rosiglitazone In Subjects With Mild To Moderate Alzheimer's Disease | 33 | Clinical Trial | Clinical |
| Plasma microRNA biomarker detection for mild cognitive impairment using differential correlation analysis - GSE90828 | 23 | Observational | Clinical |
| PREVENT Dementia Research Programme | 700 | Longitudinal | Clinical, Genomics, Imaging |
| RNAseq in Alzheimer's Disease patients - GSE53697 | 17 | Genetic | Omics (single mode) |
| RNA-sequencing of cells derived from dermis and postmortem dura mater from the same subject | 1 | Genetic | Omics (single mode) |
| Rosiglitazone (Extended Release Tablets) As Adjunctive Therapy For Subjects With Mild To Moderate Alzheimer's Disease | 1496 | Clinical Trial | Clinical |
| Rosiglitazone (Extended Release Tablets) As Adjunctive Therapy In Subjects With Mild To Moderate Alzheimer's Disease | 862 | Clinical Trial | Clinical |
| Rosiglitazone (Extended Release Tablets) As Monotherapy In Subjects With Mild To Moderate Alzheimer's Disease | 1468 | Clinical Trial | Clinical |
| Scottish Medical Imaging (SMI) Research Dataset | 999999 | Observational | Clinical, Imaging |
| Single cell RNA-seq data derived from early-onset AD cases and controls | 16 | Case-control | Omics (single mode) |
| Single cell RNA-seq data derived from MAPT carriers and controls | 16 | Genetic | Omics (multi-modal) |
| Synthetic EPAD dataset | 200 | Observational | Synthetic |
| The Airwave Health Monitoring study | 55000 | Observational | Clinical, Omics (at least one mode) |
| The Caerphilly Prospective Study | 2959 | Longitudinal | Clinical |
| The DAC Early Detection Program - Indiana University | 0 | Observational | Clinical |
| The English Longitudinal Study of Ageing | 12099 | Clinical Trial | Clinical, Omics (at least one mode) |
| The GERAS II Study | 578 | Observational | Clinical |
| The GERAS Study - EU | 1497 | Observational | Clinical |
| The GERAS Study - Japan | 553 | Observational | Clinical |
| The GERAS Study - US | 1198 | Observational | Clinical |
| The Incidence of Cognitive Impairment in Cohorts with Longitudinal Evaluation-PD (ICICLE-PD) | 318 | Longitudinal | Clinical, Genomics, Imaging |
| The SleepQuest Data set | 100 | Observational | Clinical |
| Whitehall II | 10308 | Longitudinal | Clinical |
| Whole-blood RNA-seq data derived from C9orf72+ FTD spectrum cases and controls | 114 | Case-control | Omics (single mode) |
